# National Confederation of State Medical Examining and Licensing Boards

**Published:** 1911-04

**Authors:** 


### National Confederation of State Medical Examining and Licensing Boards

THE twenty-first annual meeting of the National Confederation of State Medical Examining and Licensing Boards was held at the Congress Hotel, Chicago, February 28, 1911.

The president, Dr. Joseph C. Guernsey, Philadelphia, was in the chair.

An address of welcome was delivered by Dr. George W. Webster, Chicago. Dr. Lee H. Smith, of Buffalo, responded.

President Guernsey delivered an address on medical licensure.

### FLEXNER REPORT.

Mr. Abraham Flexner, of New York City, in his report to the confederation pointed out that the educational prerequisite must be defined, and the board authorised to insist upon it as an educational preliminary. The enforcement of even the four-year high school standard will so far clean up the medical field that the state boards will at once be relieved of the duty of dealing with actually disreputable schools. The examination for licensure is the lever with which the entire field may be lifted; for the power to examine is the power to destroy. A model state board law must guard the following points: The membership of the board must be drawn from the best elements of the profession including those engaged in teaching; the board must be armed with the authority and machinery to institute practical examinations, to refuse recognition to unfit schools, and to insist upon such preliminary educational standards as the state's own educational system warrants; finally it must be provided either by appropriation or by greatly increased fees with funds adequate to perform efficiently the functions for which it was created.

The committee to which was referred the Flexner report, composed of Drs. W. J. Means, N. P. Colwell, and Henry Beates, made the following report which was adopted.

As to the recommendations made in Mr. Flexner's paper, they concern subjects which have frequently been presented and discussed at meetings of this confederation as well as of the Association of American Medical Colleges and of the Council on Medical Education. We therefore recommend that most careful consideration be given to all the suggestions made and that the three special recommendations regarding preliminary education, refusal to recognise poorly equipped schools and practical examinations be adopted.

### COULD THE STATE CONTROL AND CONDUCT MEDICAL COLLEGES MORE EFFICIENTLY THAN CORPORATIONS AND PRIVATE INDIVIDUALS?

Dr. Charles William Dabney, president of the University of Cincinnati, answered this question in the affirmative and said the chief argument for public supported institutions in a democracy is the necessity for making them accessible to all classes of citizens on equal terms. To exclude the poor from the opportunities of the highest education or to educate them only at the bounty of the wealthy is utterly repugnant to our democratic ideals.

THE DUTY OF THE STATE IN THE CONTROL OF MEDICAL COLLEGES.

Mr. Abraham Flexner advocated state control of medical colleges and suggested that the control be indirect in this manner: First, that the state shall determine the date of registration of each medical student by setting a standard of preliminary education and permitting the student to begin to count his time of medical study only after the state has declared his preliminary education completed; second, that the state shall require a minimal period of medical study in a properly equipped medical school; third, that the state shall register for practice only such as give evidence of having obtained a certain minimal knowledge of medicine as determined by examination and practical tests.

Dr. Henry A. Christian, Boston, stated that Mr. Flexner's ideas represented an extension of what state boards are striving for and in some cases very nearly attained. He emphasised particularly the warning sounded by Mr. Flexner against any attempt to dictate to medical colleges details of methods of instruction, curriculum, etc.

Dr. Lee H. Smith, Buffalo, said state control must mean state support. The city of Buffalo has earnestly tried to enlist state support in its proposed new university. That has seemed to be difficult, but with the arousal of public opinion, public interest, public pride in the university, the state has been interested and has taken hold of the proposition and it proposes to establish and will endeavor to maintain a university that will meet with the approval even of Mr. Flexner.

Hon. Charles Alling, Jr., Chicago, spoke of state support and control of medical education and said that if the courts will sustain as a requirement for an examination a diploma from an institution of the standards upheld by the association of American Medical Colleges they should for the same reason sustain a law providing that the degree of M.D. shall be given only by medical colleges in good standing with the state board of medical registration and examination, an official body created by and acting for the state to secure good medical service for all the people.

THE ADVANTAGES TO MEDICAL COLLEGES OF STATE CONTROL.

Dr. Arthur Dean Bevan, Chicago, said proper state control would mean a great deal and be of great advantage to the medical colleges which are doing good work and developing along scientific lines. He believes that if every state board represented in the confederation would attempt to get the profession of its own state back of it and make proper state control effective, that within five years American medicine could be placed in a position where we could say that the American medical student is obtaining the proper medical education; that he has had a proper preliminary and medical education and some practical hospital experience before he is allowed to practise medicine.

### SOME EVILS IN THE PRESENT SYSTEM OF MEDICAL EDUCATION.

Dr. F. C. Waite, Cleveland, Ohio, said the evils of overproduction of medical graduates are self evident. First, the field is overstocked; consequently the average income is reduced; second, from this the result must be that the more able members of following generations will shun the profession and its recruits must come from the mediocre and the general tone of the profession will thereby diminish; third, the average lack of income from reputable and ethical practice of the profession will lead many into quackery and charlantry and thereby still more reduce the dignity and respectability of the profession which again will divert the tendencies of able young men from the profession.

### STATE AID IN THE SECURING OF MORE FULL TIME SALARIED TEACHERS

Dr. Frank Winders, Columbus, Ohio, said full-time, salaried men are indispensable to the proper teaching of the first and second year students of the medical curriculum. This plan is now in practice in every first-class medical school. The true purpose of a medical college is found only in teaching the science and art of medicine to the end that its students and graduates may be best equipped to prevent and relieve the ills and accidents of mankind. If medical schools were aided and controlled by the state the same excellent conditions would obtain in all schools as are found in our state universities.

### STATE CONTROL OF MEDICAL EDUCATION AND ENFORCEMENT OF THE MEDICAL PRACTICE ACT.

Dr. Edward Cranch, Erie, Pa., said the method of full control would be suitable for one or two colleges in each state and if others wish to organise they can, under a modified control, be rendered equally efficient and yet preserve that initiative which will develop new and better methods for the future. In this way examining and licensing boards will find their work supported by the colleges instead of being antagonised and criticised as at present. This alone furnishes a good argument for at least a modified state control.

### RESULT OF STATE CONTROL OF MEDICAL COLLEGES ON PROFESSIONAL EFFICIENCY.

Dr. Royal S. Copeland, New York City, said when one considers the really ideal condition as regards medical college supervision he thinks of the superior advantages of federal control with uniform standards of admission, graduation and licensure.

This, however, is impossible at present and perhaps would be unwise. Of necessity low standards will prevail in pioneer and undesirable sections of the country and a federal law, in striking an average for the country at large, would raise a much higher standard than could obtain in many of the individual states. State control is the present logical solution of the problem. In the matter of medical colleges private control undoubtedly results in abuses and a lower standard than should be tolerated at the present time. Certainly private control must mean lack of uniformity. State control has all the benefits of federal control if there are reciprocal relations between the states. This reciprocal relationship would be much easier to realise were there uniformity in the college curricula as regards the essentials of medical education. The greatest argument for state supervision is the protection of the people against charlatans, quacks, and poorly educated practitioners.

Dr. James A. Eagan, Springfield, Illinois, read a paper entitled "Some Thoughts on the Supervision of Medical Colleges and the Conducting of State Examinations."

The following officers were elected: President, Dr. Charles A. Tuttle, New Haven, Conn.; first vice-president, Dr. James A Egan, Springfield, Illinois; second vice-president, Dr. A. B. Brown, New Orleans, La.; secretary-treasurer, Dr. George H. Matson, Columbus, Ohio; executive council, Dr. N. R. Coleman, Columbus, Ohio, chairman; Dr. Charles H. Cook, Natick, Mass.; Dr. Joseph C. Guernsey, Philadelphia; Dr. W. Scott Nay, Underhill, Vermont; Dr. James A. Duncan, Toledo, Ohio.

Chicago, Illinois, was selected as the place for holding the next annual meeting.

